# Heat shock proteins and cancer therapy: The trail grows hotter!

**DOI:** 10.18632/oncotarget.294

**Published:** 2011-06-11

**Authors:** Elizabeth A. Repasky, John R. Subjeck

**Affiliations:** Depts. of Immunology and Cell Stress Biology, Roswell Park Cancer Institute, Buffalo NY 14263

The physiological properties of the heat shock response were initially studied over three decades ago in Drosophila based on the observation that it had protective functions against teratogenic agents and heat induced animal death. While initially believed to be unique to flies, it was rapidly observed to be universal response common to all living organisms. Early mammalian studies (reviewed in ref. [1]) focused on the physiological role of heat shock proteins or HSPs in protecting the organism against heat, a phenomenon called thermotolerance. It was determined that HSPs were able to protect cellular proteins and therefore cells from proteotoxic stress such as heat shock. This general function of HSPs are not restricted to stress protection, but is an essential element in the natural operation of cellular machinery which can also lead to protein damage under non-stress conditions in a protein concentrated intracellular environment. Such interactions of the HSP with cellular proteins would protect the substrate protein from the damaging effects of heat and other proteotoxic conditions as well as to aid in the clearance of some damaged proteins via degradation. In cancer research, HSPs were initially perceived to play a role protecting tumors from hyperthermia when applied in multiple treatments, i.e. HSPs induced initially would protect the tumor from later treatments [2]. It took many more years to begin to appreciate that there are other and potentially more significant roles for HSPs in cancer. For example, it was recognized long ago that interference with HSP90, could revert tumor cells from a transformed to a normal phenotype due to the role of HSP90 in facilitating the functions of tyrosine kinases necessary in cell transformation. The ability to block HSP90 function has since been aggressively pursued as a cancer therapy using specific HSP90 inhibitors such as geldanamycin [3]. Later studies revealed the additional and more general role for HSPs in cancer. All proliferating cells require an increase in HSP chaperone concentrations due to an increase in macromolecular activities that occur compared to quiescent cells. This increase in activity can be expected to lead to an increase in various phenomena such as free radical formation and related protein damage in proliferating cells. In addition to this, the increased accumulation of mutated proteins expected in cancer also poses a further likelihood for cell damage compared to normal cells. Combined, these phenomena can result in the protein damage signals leading to the enhanced heat shock response and HSP levels, protecting the cancer cells from the accumulation of misfolded proteins. Thus, cancer cells can have an already activated heat shock response, at least in part, relative to normal cells. While a heat shock response can be robust, the pre-stress activated response in cancer suggests that cancer cells would be less capable of mounting a further strong response relative to normal cells. Therefore, additional proteotoxic stress such as hyperthermia may be more toxic to cancer cells relative to normal cells. A new paper by Neznanov et al [4] examines this hypothesis. It proposes that that proteotoxic stress such as hyperthermia might be more effective in killing cancer relative to normal tissues since a level of heat shock response is already partially occupied in cancer cells. Neznanov et al examined the combinations of three forms of proteotoxic stress, such as hyperthermia, puromycin treatment, and bortezomib treatment (which blocks protein degradation thereby increasing the accumulation of damaged proteins that would otherwise be eliminated). They show that these treatments caused substantial control of multiple myeloma cell growth, in vitro and in vivo. The increased cell death in cancer cells was found to be largely due to a p53-independent apoptotic process. This study provides tantalizing evidence that cancer cells are already addicted to the heat shock response for their own survival as hypothesized. The same group has previously used another approach to achieve a similar outcome. They have demonstrated that the inhibition of HSP expression or function through either inhibitors of the transcription factor heat shock factor 1or inhibitors of HSP70 function can also be toxic to cancer cells, again based on the same rationale, i.e. the enhanced need for HSPs in cancer cells [5]. Therefore the heat shock response has important survival functions in the development and progression of cancer. Importantly, studies from the Calderwood group also connect the heat shock response with carcinogenesis [6]. This information introduces new targets to exploit in cancer therapy. It is uplifting to see the very early and pioneering studies on the protective functions of HSPs in Drosophila being now applied to cancer therapy.
